# Short-term anti-remodeling effects of gliflozins in diabetic patients with heart failure and reduced ejection fraction: an explainable artificial intelligence approach

**DOI:** 10.3389/fphar.2023.1175606

**Published:** 2023-06-09

**Authors:** Marco Mele, Paola Imbrici, Antonietta Mele, Maria Vittoria Togo, Giorgia Dinoi, Michele Correale, Natale Daniele Brunetti, Orazio Nicolotti, Annamaria De Luca, Cosimo Damiano Altomare, Antonella Liantonio, Nicola Amoroso

**Affiliations:** ^1^ Department of Pharmacy-Drug Sciences, University of Bari, Bari, Italy; ^2^ University Hospital Policlinico Riuniti, Foggia, Italy; ^3^ Department of Medical and Surgical Sciences, University of Foggia, Foggia, Italy; ^4^ National Institute of Nuclear Physics, Section of Bari, Bari, Italy

**Keywords:** heart failure, gliflozins, sodium-glucose cotransporter 2 inhibitor, explainable artificial intelligence, machine learning

## Abstract

**Introduction:** Sodium-glucose cotransporter type 2 inhibitors (SGLT2i), gliflozins, play an emerging role for the treatment of heart failure with reduced left ventricular ejection fraction (HFrEF). Nevertheless, the effects of SGLT2i on ventricular remodeling and function have not been completely understood yet. Explainable artificial intelligence represents an unprecedented explorative option to clinical research in this field. Based on echocardiographic evaluations, we identified some key clinical responses to gliflozins by employing a machine learning approach.

**Methods:** Seventy-eight consecutive diabetic outpatients followed for HFrEF were enrolled in the study. Using a random forests classification, a single subject analysis was performed to define the profile of patients treated with gliflozins. An explainability analysis using Shapley values was used to outline clinical parameters that mostly improved after gliflozin therapy and machine learning runs highlighted specific variables predictive of gliflozin response.

**Results:** The five-fold cross-validation analyses showed that gliflozins patients can be identified with a 0.70 ± 0.03% accuracy. The most relevant parameters distinguishing gliflozins patients were Right Ventricular S'-Velocity, Left Ventricular End Systolic Diameter and E/e' ratio. In addition, low Tricuspid Annular Plane Systolic Excursion values along with high Left Ventricular End Systolic Diameter and End Diastolic Volume values were associated to lower gliflozin efficacy in terms of anti-remodeling effects.

**Discussion:** In conclusion, a machine learning analysis on a population of diabetic patients with HFrEF showed that SGLT2i treatment improved left ventricular remodeling, left ventricular diastolic and biventricular systolic function. This cardiovascular response may be predicted by routine echocardiographic parameters, with an explainable artificial intelligence approach, suggesting a lower efficacy in case of advanced stages of cardiac remodeling.

## 1 Introduction

Heart failure (HF), a clinical syndrome due to increased intra-cardiac pressure and/or inadequate cardiac output, is one of the major causes of death and hospitalization in diabetic patients (Shah et al., 2015; Koudstaal et al., 2017; McDonagh et al., 2021). The cornerstone of medical treatment for HF is represented by beta-blockers (BB), angiotensin-converting enzyme inhibitors (ACEi), angiotensin receptor-neprilysin inhibitors (ARNI), and mineralocorticoid receptor antagonists (MRA) (McDonagh et al., 2021; Mascolo et al., 2022).

Gliflozins, which act as sodium-glucose cotransporter type 2 inhibitors (SGLT2i), are a new class of blood glucose-lowering medications that block renal glucose reabsorption in the proximal tubule, thereby increasing urinary glucose excretion and improving glycemic control (Steen and Goldenberg, 2017). Initially approved for the treatment of the type 2 diabetes mellitus (T2DM), SGLT2i provided a reduction in cardiovascular (CV) outcomes (McDonagh et al., 2021). The EMPA-REG OUTCOME trial with empagliflozin, the CANVAS Program with canagliflozin, the DECLARE-TIMI 58 with dapagliflozin, and real-life data from the CVD-Real Study demonstrated that SGLT2i may reduce CV events and improve both mortality and hospitalization rates in diabetic patients (Zinman et al., 2015; Kosiborod et al., 2017; Neal et al., 2017; Wiviott et al., 2018). More recently, results from the EMPEROR-Reduced trial with empagliflozin and results from DAPA-HF trial confirmed the reduction of CV death, HF hospitalization, and worsening in patients with reduced left ventricular ejection fraction (HFrEF, left ventricular ejection fraction (LVEF) ≤ 40%), regardless of the presence or absence of diabetes (Packer et al., 2017; McMurray et al., 2019). For these reasons, in the HF 2021 European Society of Cardiology Guidelines, gliflozins have been introduced for the treatment of patients affected by HFrEF independently of diabetes (McDonagh et al., 2021).

Despite robust evidence for the CV benefit of SGLT2i, some important questions remain unanswered. Among them, the effect on cardiac remodeling and function, in particular with regards to the right ventricle (RV), has not been completely established. Moreover, the best timing to start gliflozin treatment and parameters possibly predicting clinical response have not been sufficiently evaluated.

Explainable artificial intelligence (XAI) and machine learning (ML) methods have achieved remarkable progress, and their use has increased significantly over the last few years in CV medicine (Mathur et al., 2020; Romiti et al., 2020; Cheng et al., 2021; Togo et al., 2023). Several potential applications of ML in HF have been described, including risk stratification, early diagnosis, optimal indications for specific treatments, and understanding drugs molecular mechanisms of action (Iborra-Egea et al., 2019; Lorenzoni et al., 2019; Bayes-Genis et al., 2021; Sermesant et al., 2021; Yang et al., 2022).

We, therefore, aimed at applying an ML approach in order to evaluate the short-term CV effect of gliflozins in diabetic patients with HFrEF in terms of LV and RV remodeling and function detected with echocardiography, ultimately disclosing the echocardiographic variables as more effective in predicting the CV response to gliflozins.

## 2 Materials and methods

### 2.1 Data sources

**FIGURE 1:**
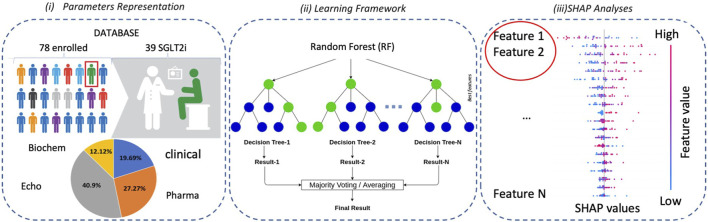
Overview of the methodological approach: (i) the enrolled patients are represented by a set of clinical parameters; (ii) a Random Forest (RF) classifier is then used for classification purposes; and (iii) finally, an explainability analysis is carried out by means of a SHAP analysis.

From 28 January 2019 to 9 March 2021, we enrolled 78 consecutive diabetic outpatients followed up for HFrEF at Policlinico Riuniti University Hospital (Foggia, Italy). Each patient received evidence-based pharmacological treatment according to the European Society of Cardiology guidelines for treatment of chronic heart failure (McDonagh et al., 2021), clinical decision, individual tolerance, and contraindications. Thirty-eight patients were also treated with SGLT2i. Clinical data, echocardiographic, biochemical, and pharmacological parameters (for a total of 66 parameters) were recorded from all patients at baseline and at 4–6 month follow-up (Figure 1). Based on the European Society of Cardiology Guidelines for the Management of HF (McDonagh et al., 2021), such a range of time is considered appropriate to assess the short-term efficacy associated with drug administration. Echocardiographic data analysis was performed by the same fully accredited operator using ultrasound device Philips EPIQ 7c (Philips, Amsterdam, Netherlands). Patients without diabetes and with preserved or mid-reduced left ventricle ejection fraction were excluded from the analysis. The informative content provided by the available features was then exploited by means of a random forest (RF) classifier and a SHAP (SHapley Additive exPlanations) explainability analysis. The framework is schematically presented in Figure 1.

### 2.2 Random forest classification and feature importance

To evaluate the informative content of the available clinical features, a RF classifier was employed (Biau and Scornet, 2016). This supervised learning algorithm exploited an ensemble of tree classifiers, each one grown with different features and diverse partitioning of the training set, to achieve a statistically robust classification. In this respect, a subset of the available training examples was first randomly selected to set the model parameters, while those left out, namely, out-of-bag observations, were used instead to evaluate the model performances and minimize overfitting; the square root of available features was randomly selected to increase the branching of the decisional tree at each split, and the operation was repeated until all available examples were assigned to a given class. This procedure was optimized by using Gini’s index, a metric assessing the node purity, i.e., the separation between the classes. The goal was to determine determining the optimal cuts by discriminating the classes on the basis of the available features. In this respect, several different tree classifiers were trained to achieve statistical robustness, with this task intrinsically related to the number observations and of the randomly selected features. Final decisions were taken by majority vote, across all generated trees.

The number of trees within the forest and the number of features to pick represented the most important parameters to be tuned for the RF model. The internal validation provided by out-of-bag observations, along with the ease of tuning, made this modeling approach a first choice for many different applications. Moreover, it was worth saying that RF was capable of achieving state-of-the-art performances that were often comparable even with more sophisticated and computationally demanding algorithms, such as neural networks. Finally, RF provided an embedded way to measure feature importance, thus providing a basic but very effective way to understand which features best contributed to the model’s accuracy.

Each time a feature was used to split a node, Gini importance measured the purity of the node, i.e., how well that feature was able to separate the available classes. This measure was averaged across the forest and used to rank the importance of each feature based on its impact on node purity. However, such an approach returned an overall view of the feature contribution to the model accuracy, and no information could be derived about a single decision. Noteworthy, the XAI paradigm was employed to make the classifier’s decision more transparent and desirable.

### 2.3 Explainability according to Shapley’s values

In the present work, the Shapley paradigm was adopted (Shapley, 2016) to derive classification models that are explainable and easy to interpret. Such approach was based on the idea that all available features behaved like the players of a team (i.e., the classifier), whose final score summarized the goals of each player. Likewise, the contribution of each feature was modeled through a linear relationship so that the final score assigned by the model to each prediction could be explained as the sum of the contributions of all the features. Thus, this framework not only provided a global feature importance evaluation (as RF already did) but, more importantly, gave the option to “locally” inspect the model decisions and explain how they were reached. According to the SHAP (SHAP website: https://shap.readthedocs.io/en/latest/index.html) package implemented in Python, we evaluated for each feature *j* the *SHAP* value:

$$SHAP_{j}\left(o\right)=\underset{c:j\in c}{\sum }{\left[\left|c\right|\times \left(\begin{array}{l} F\\ \left|c\right| \end{array}\right)\right]}^{-1}\left[p_{c}\left(o\right)-p_{c-j}\left(o\right)\right],$$

with *F* being the total number of input features, *c* a subset of the features, and |*c*| their number; an observation was a vector **o** whose components were the input features, *p*_*c*_(**o**) the prediction yielded by the features in *c,* and *p*_*c-j*_(**o**) the prediction obtained without the *j* feature. Thus, the importance of each feature on the model prediction was evaluated by averaging all possible differences.

### 2.4 Performance evaluation

The presented machine learning and explainability analyses were carried out using cross-validation to ensure unbiased performance and Shapley value estimates. In particular, we adopted a repeated five-fold cross-validation for analyses concerning the whole set of patients to assess the SGLT2i effectiveness. For this classification task, the classes to predict were the one of patients treated with gliflozins against the one including patients undergoing standard treatment. Instead, a leave-one-out approach was used for the cohort of only treated patients whose size was limited; here, the labels distinguished the patients treated with gliflozins from those who responded to from treatment in a different way from those who were standardly treated. To this aim, we performed several cross-validation rounds of the previous task and counted the number of times when patients treated with gliflozins were correctly classified and when they were misclassified; this measure yielded an operative definition of “responders” and “not-responders.”

Classification performance was evaluated in terms of accuracy, sensitivity, and specificity:

$$Accuracy=\frac{TP+TN}{TP+TN+FP+FN}$$

$$Sensitivity=\frac{TP}{TP+FN}$$

$$Specificity=\frac{TN}{TN+FP}$$

While accuracy globally evaluated the model performance, sensitivity and specificity characterized the model behavior with respect to the positive class (patients treated with gliflozins) and the negative class (patients not treated with gliflozins). Cross-validation analyses allowed us to estimate an average value for each adopted metric, while the related uncertainties were reported in terms of standard deviations.

## 3 Results

### 3.1 Are patients treated with gliflozins distinguishable from non-treated ones? A random forest analysis

**FIGURE 2:**
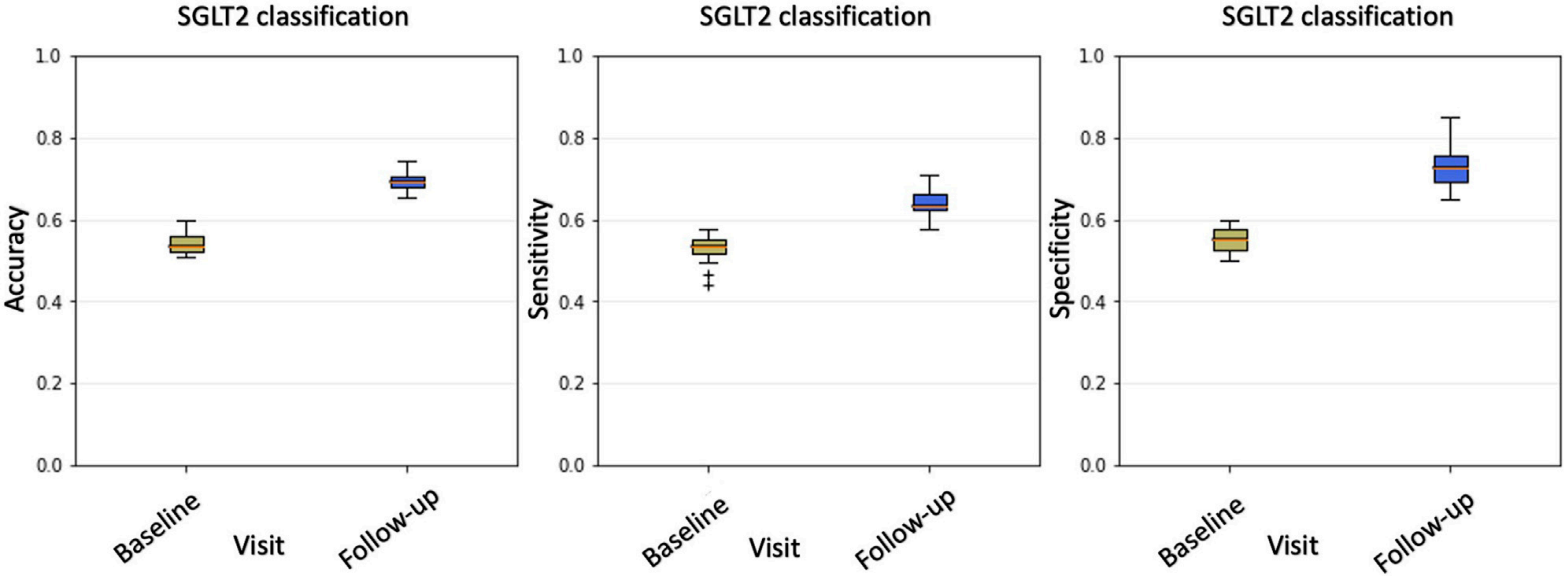
From left to right: accuracy, sensitivity, and specificity boxplots comparing baseline and follow-up classification performance. For each metric, the results obtained through 100 repeated five-fold cross-validations are reported. According to all three metrics, after gliflozin treatment, patients who were undistinguishable at baseline show significant differences.

We enrolled 78 consecutive diabetic outpatients followed up for HFrEF at Policlinico Riuniti University Hospital (Foggia, Italy), of whom 38 were treated with SGLT2i. The main demographics, clinical, and biochemical characteristics, as well as treatments, of these patients are listed in Table 1. By using an RF model, we initially evaluated, on the available cohort of 78 patients and based on clinical, echocardiographic, biochemical, and pharmacological parameters, if patients treated with gliflozins could be distinguished from non-treated ones at baseline and at 4–6 month follow-up. We, therefore, trained two different RF models to discriminate patients treated with gliflozins (positive class “1”) from patients undergoing standard treatment (negative class “0”): the first reporting baseline features (i.e., before treatment); the second concerned with follow-up (i.e., after treatment with gliflozins). A synoptic view is shown in Figure 2.

**TABLE 1:** List of patient features included in the database.

Clinical	
Age (years)	67.9 ± 1
Male (%)	84.6
Body weight (kg)	85.4 ± 2
Time from diabetes mellitus diagnosis (years)	9.1 ± 0.6
SBP (mmHg)	118.7 ± 2.4
DBP (mmHg)	71.5 ± 1.2
Heart rate (bpm)	69.7 ± 1.4
NYHA class ≥2 (%)	96.1
Medical history	
Arterial hypertension (%)	75.6
Anemia, Hb < 11 (%)	6.4
COPD (%)	21.8
Atrial fibrillation/flutter (%)	39.7
Medications	
ACEi/ARB/ARNI (%)	89.7
Beta-blockers (%)	92.3
MRA (%)	47.4
Diuretics (%)	89.7
Statins/fibrate (%)	88.5
Allopurinol (%)	37.2
Ivabradine/ranolazine (%)	47.4
Digoxin (%)	15.4
Amiodarone (%)	10.2
Antiplatelet drugs (%)	28.2
Anticoagulant drugs (%)	51.3
Warfarin/acenocoumarol (%)	16.6
Apixaban (%)	12.8
Dabigatran etexilate (%)	7.7
Rivaroxaban (%)	12.8
Edoxaban (%)	1.2
Other antidiabetic drugs (%)	62.8
Insulin (%)	38.5
Laboratories	
HbA1c (%)	8.0 ± 0.2
SCr (mg/dL)	1.1 ± 0.03
MDRD-based eGFR (mL/min)	72.5 ± 2.3
CRP (mg/L)	2.9 ± 0.3
Ca-125 (U/mL)	22.4 ± 5.4
NT-pro-BNP (pg/mL)	1340.6 ± 334.5
CKD stage ≥2 (%) eGFR (mL/min)	91.02 72.5 ± 2.3
ESR (mm/h)	21.37 ± 2.7
Echocardiographic	
LVEDD (mm)	56.7 ± 0.8
LVESD (mm)	47.6 ± 0.9
IVS (mm)	12.0 ± 0.2
LVPW (mm)	10.8 ± 0.2
LV Mass (g)	271.8 ± 8.3
RWT (ratio)	0.4 ± 0.01
LVMI (g/m^2^)	139.1 ± 3.8
LVEF (%)	38.9 ± 0.9
EDV (mL)	151.9 ± 7.6
ESV (mL)	97.9 ± 6
MR ≥ 2 (%)	33.3
LAD (mm)	44.3 ± 0.8
LA Area (mm^2^)	22.7 ± 0.7
LAVI (mL/m^2^)	36.8 ± 1.6
LAV (mL)	72.8 ± 3.5
TR ≥ 2 (%)	19.2
TAPSE (mm)	18.7 ± 0.4
RV S’ (cm/s)	10.7 ± 0.3
sPAP (mmHg)	30.5 ± 1.0
E/A	1.7 ± 0.3
E/è	14.2 ± 1
E wave (cm/s)	80.2 ± 3.6
A wave (cm/s)	86.6 ± 3.6
EDT (ms)	206.1 ± 11.9
è (cm/s)	7.1 ± 0.2
s’ (cm/s)	7.6 + 0.6
LV GLS (%)	−10.4 ± 0.3

Systolic blood pressure- SBP, Diastolic blood pressure- DBP, beats per minute- BPM, New York Heart Association- NYHA, chronic obstructive pulmonary disease- COPD, angiotensin-converting enzyme inhibitors- ACEi, angiotensin II, receptor blockers- ARB, angiotensin receptor-neprilysin inhibitors- ARNI, mineralcorticoid receptor antagonist- MRA, glycated hemoglobin- HbA1c, serum creatinine- SCr, Modification of Diet in Renal Disease- MDRD, estimated glomerular filtration rate- eGFR, C-reactive protein- CRP, plasma N-terminal pro-brain natriuretic peptide- NT-pro-BNP, chronic kidney disease- CKD, erythrocyte sedimentation rate- ESR, left ventricular end diastolic diameter- LVEDD, left ventricular end-systolic diameter- LVESD, inter-ventricular septum thickness- IVS, left ventricular posterior wall thickness- LVPW, left ventricular mass- LV, mass, relative wall thickness- RWT, left ventricular mass index- LVMI, left ventricular ejection fraction- LVEF, end diastolic volume- EDV, end systolic volume- ESV, mitral regurgitation- MR, left atrial diameter- LAD, left atrium area- LA area, left atrium volume index- LAVI, left atrium volume- LAV, tricuspid regurgitation- TR, tricuspid annular plane systolic excursion- TAPSE, right ventricular systolic excursion velocity- RV S′, systolic pulmonary artery pressure- sPAP, E wave deceleration rate- EDT, left ventricular global longitudinal strain- LV GLS.

At the baseline, classification accuracy was 0.52±0.03 , a value comparable with chance for a binary classifier. The same considerations held true for sensitivity 0.53±0.03 and specificity 0.52±0.03 . As far as follow-up was concerned, classification performance showed a significant improvement for all the metrics: accuracy 0.70±0.03 , specificity 0.73±0.05 and sensitivity 0.65±0.04 . Therefore, according to the three metrics, after gliflozins treatment, patients that were undistinguishable at the baseline showed significant differences. As the only difference between the two cohorts at the follow-up was the clinical treatment, it is reasonable to conclude that gliflozins affected the patients’ conditions differently from standard treatments.

**FIGURE 3:**
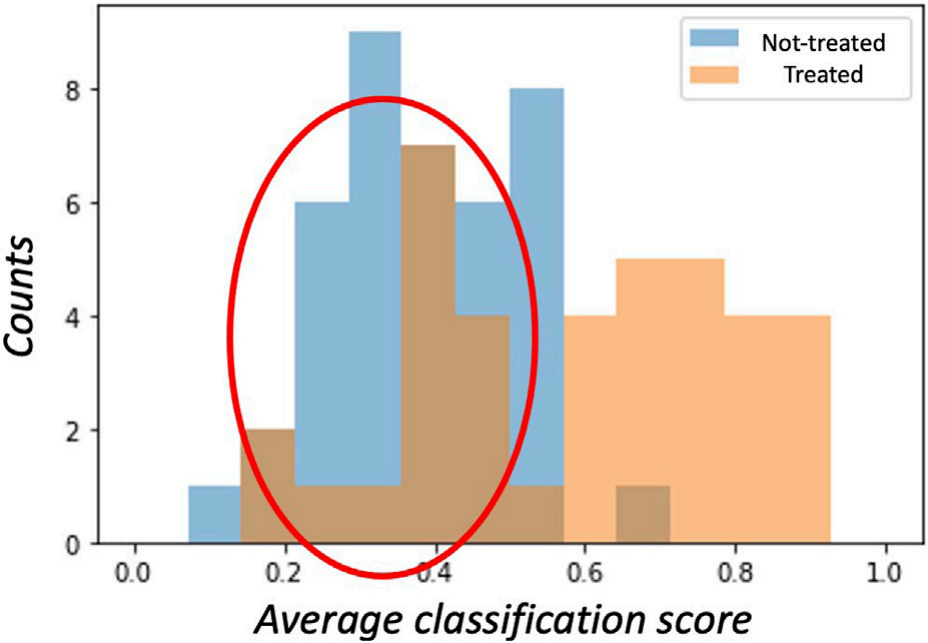
The histogram of average cross-validation scores shows how, on average, treated patients (class ‘1’) have scores distributed towards the right hand-side 1 limit, while non-treated (class ‘0’) patients have scores distributed towards the left hand-side 0 limit. Interestingly, treated patients are often misclassified (red circle) as having scores lower than 0.5. These patients are treated with gliflozins but are erroneously assigned to the ‘0’ class.

The unbalancing between sensitivity and specificity suggests that the classification model does not equally perform on the two classes; in particular, higher specificity values should indicate that the majority of misclassified examples belong to the class of treated patients. A visual confirmation was obtained by examining the histogram of the average cross-validation scores shown in Figure 3.

A subject belonging to the ‘1’ class (treated) is correctly classified if the model assigns a score greater than 0.5; analogously, a score lower than 0.5 is assigned to a patient in the ‘0’ class (not treated). Thus, for a perfect model, the score distributions should ideally be separated and show the so-called bathtub distribution. In our case, the distribution of scores among treated patients showed a huge tail below the 0.5 threshold. Accordingly, within the SGLT2i treated group, we labeled the 16 patients responding to treatment as ‘responders’ to distinguish them from the remaining 22 called ‘not-responders.’ Conversely, only few examples of the non-treated patients presented scores exceeding the threshold on average.

### 3.2 What are the features that allow us to distinguish treated from untreated patients at FU? A SHAP explanation and analysis

**FIGURE 4:**
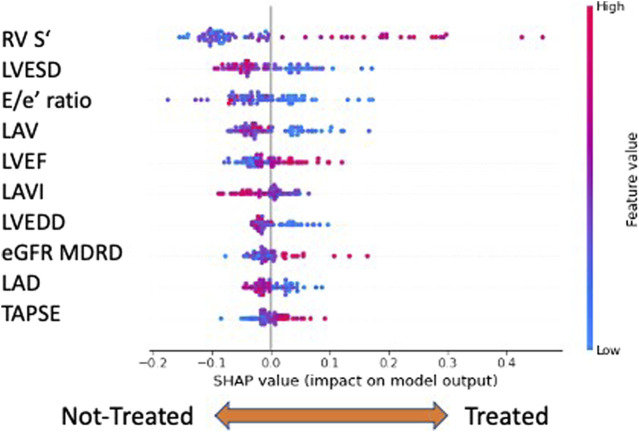
Global explainability: the top ten features are ranked (from top to bottom) according to their importance. The horizontal axis evaluates the feature impact on the model decisions: right positive SHAP values contribute to assigning observations to the class of treated patients ‘1’, left negative values to non-treated ones. In addition, high/low feature values are color coded. For example, high ‘RV S’ values and low ‘LVESD’ characterize treated patients. Legend: RV S′, right ventricular systolic excursion velocity; LVESD, left ventricular end-systolic diameter; E/e’, E velocity/e’ velocity ratio; LAV, left atrium volume; LVEF, left ventricular ejection fraction; LAVI, left atrium volume index; LVEDD, left ventricular end-diastolic diameter; eGFR MDRD, estimated glomerular filtration rate MDRD (Modification of Diet in Renal Disease); LAD, left atrial diameter; and TAPSE, tricuspid annular plane systolic excursion.

Previous results demonstrated that the gliflozin treatment made patients much easier to distinguish from baseline to follow-up. Thus, we wondered which features were driving the classifier’s decisions. A first answer to this question could be given from a global perspective by inspecting the average SHAP values of the classification model (Figure 4). In our model, the most important features able to discriminate between treated and untreated patients were the echography-related ones, listed in order of relevance in Figure 4. Global explainability ranks the features according to their average importance in the model’s decisions. As a difference to classical feature importance rankings, the SHAP paradigm allows one to directly interpret how features contribute. For example, the most important feature for the RF model was the ‘RV systolic excursion velocity’ (RV S^’^) variable. High values of this feature are strong predictors of survival for HFrEF patients. Interestingly, by visual inspection of the SHAP graph, it was evident that high RV S^’^ values characterized treated patients. On the contrary, the second important variable, left ventricular end systolic diameter (LVESD), had low values for treated patients and high values for non-treated ones, as expected by an efficacious HF treatment.

### 3.3 Which are the features that allow us to distinguish responders from non-responders at baseline?

**FIGURE 5:**
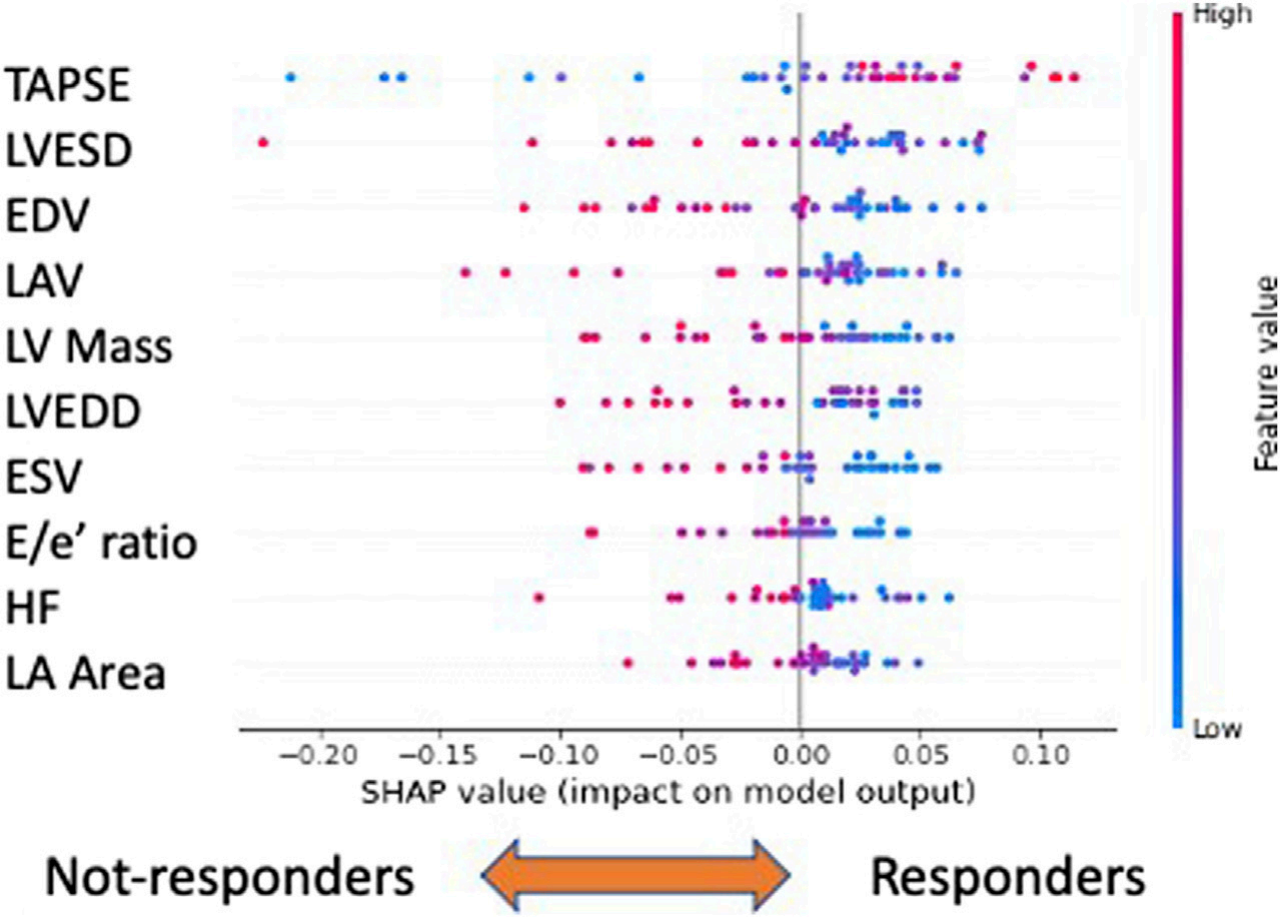
Explainability analysis of responder *versus* non-responder classification. In this case, the ten most important features are shown. The top three performers are “TAPSE,” “LVESD,”and “EDV”: low values of “TAPSE” and high values of the remaining features characterize not-responders. Legend: TAPSE, tricuspid annular plane systolic excursion; LVESD, left ventricle end systolic diameter; EDV, end diastolic volume; LAV, left atrium volume; LV Mass, left ventricular mass; LVEDD, left ventricle end diastolic diameter; ESV, end systolic volume; E/e’, E velocity/e’ velocity ratio; HR, heart rate; and LA area, left atrial area.

Previous analyses demonstrated that, based on clinical features, treated patients could be distinguished by RF. Nevertheless, there were some misclassified cases (labeled as non-responders) especially among the treated patients. Thus, we considered only the 38 treated patients representing the ‘1’ class and distinguished those misclassified among the patients on average correctly classified (responders) (average classification score greater than 0.5). As the number of such cohorts was limited, for the subsequent analyses, we adopted a leave-one-out cross-validation framework. We observed that using only baseline features, it was possible to accurately (74%) predict which subjects would have successfully responded to gliflozins treatment. Even in this case, we performed a global SHAP explanation of the model (Figure 5).

Within the global feature importance evaluation, the most important feature to distinguish responders from non-responders was tricuspid annular plane systolic excursion (TAPSE), a reliable index of the right ventricle systolic function (McDonagh et al., 2021). Concerning the other variables, among the top ten, echocardiographic variables kept playing a relevant role. In general, low values of ‘TAPSE’ and high values of the remaining features, such as heart rate (HR), left ventricular end systolic diameter, end diastolic volume (EDV), left ventricular mass (LV Mass), and left atrial volume (LAV), characterize non-responders.

## 4 Discussion

In this study, we aimed at assessing the short-term effect of gliflozins on cardiac remodeling and function and revealing possible predictors of clinical response using XAI algorithms in a population of diabetic patients with HFrEF. Beyond a positive effect on LV remodeling and performance, we detected a beneficial effect on RV systolic function.

In addition to glycemic control, several mechanisms have been hypothesized to explain the beneficial CV effects of gliflozins (Verma and McMurray, 2018). Plausible assumptions included improvement in volume status, natriuresis, expansion of red blood cell mass, and myocardial energetics (Heerspink et al., 2016; Lytvyn et al., 2017; Verma and McMurray, 2018). Furthermore, a direct effect of gliflozins on LV remodeling, in particular on LV mass, volumes, and systolic and diastolic functions, has been reported (Verma et al., 2019; Salah et al., 2022). However, there was less convincing evidence on the impact of gliflozins on RV parameters. In a *post hoc* analysis, empagliflozin showed no significant impact on RV volumes or mass index in T2DM patients with coronary artery disease (Sarak et al., 2021). On the other hand, in the randomized, multicenter, double-blind, placebo-controlled trial EMBRACE-HF, empagliflozin resulted in a reduction of pulmonary artery pressures regardless of the use of diuretics, although the proportion of diabetic patients was only 18% (Nassif et al., 2021). Interestingly, our study provided evidence of the contribution of gliflozins to improving RV systolic function, as highlighted by the SHAP value obtained for RV S’ velocity. As RV dysfunction was a strong predictor of survival for HFrEF (Sun et al., 1997; Ghio et al., 2001), RV systolic function improvement may contribute to the well-known reduction of mortality and hospitalization in diabetic patients with HFrEF (Kosiborod et al., 2017; Neal et al., 2017; Wiviott et al., 2018). Distinct mechanisms of action and targets involving multiple biochemical and hemodynamic pathways have been proposed to explain the effects of gliflozins at the CV level and can also support an improvement of the RV systolic function (Scheen, 2020). Direct myocardial effects such as a reduction in myocardial stretch and inhibition of Na^+^-H^+^ exchanger 1 or a reduction in oxidative stress and low-grade inflammation (Lorenzoni et al., 2019; Packer et al., 2020; Kondo et al., 2021; Zuurbier et al., 2021) could be relevant in this regard.

Regarding the potential influence on the remodeling and ejection fraction by other drugs concomitantly used, such as MRA and digoxin, it is worth noting that in our exploratory analysis all the available features were employed to train a classification model and never a classification accuracy different from chance was achieved; this led us to conclude that the other pharmacological treatments did not significantly affect the cohort.

The metabolic efficacy of SGLT2i in patients with T2DM has been investigated; the glucose-lowering effect appeared greater in patients with a shorter duration of T2DM, better renal function, and higher levels of HbA1c (Cho et al., 2019). These clinical factors may help to predict ‘metabolic responders’ to treatment with SGLT2i. On the other hand, poor data exist about markers predicting the response in terms of CV outcomes. Vaduganathan et al. (2022) demonstrated that higher levels of stress cardiac biomarkers, in particular troponin I, soluble suppression of tumorigenesis 2 protein, and insulin-like growth factor binding protein 7, were associated with a greater relative risk reduction in terms of CV events in the CANVAS study population, namely, in diabetic patients. LVEF appeared to poorly predict CV response, to gliflozins. According to a pooled analysis of the EMPEREOR-Reduced and EMPEREOR-Preserved trials, the beneficial effect of empagliflozin was similar in patients with LVEF <25% and <65% and produced a reduced response in patients with LVEF ≥65%, in terms of major adverse cardiovascular events and hospitalizations (Butler et al., 2022). Moreover, the use of algorithms failed to predict adequately CV and renal effects of gliflozins (Tye et al., 2022).

According to our observations, at a short-term follow-up, the population of gliflozins-treated patients was clearly distinguishable from non-treated patients, as evident in the histogram of average cross-validation scores. In addition, within the gliflozin-treated group, some patients were misclassified. The explainability analysis disclosed a pool of clinical variables that allowed us to distinguish the responder vs. non-responder patients at baseline with an accuracy of 74%. Actually, eight echocardiographic parameters and the presence of a high heart rate may predict the response to gliflozins in terms of anti-remodeling effects. In more detail, the presence of LV remodeling in advanced stages, high degree of RV dysfunction, and a higher heart rate may all together predict a lower probability of a beneficial response to gliflozins. Furthermore, TAPSE is considered a reliable index of the right ventricle’s systolic function (McDonagh et al., 2021). Importantly, the right heart dysfunction may be a primitive disorder but is more often associated with left HF in advanced stages (Gorter et al., 2018). Thus, low TAPSE values characterise patients with an advanced-stage HF and biventricular dysfunction. In other words, it appeared that patients with an overall lower TAPSE, higher LV mass and volumes, worse LV diastolic function, the presence of AF, and a higher HR could likely respond less to therapy with gliflozins.

Even if highly recommended as BB, ACEi/ARNI, or MRA for patients affected by HFrEF, including diabetic patients, there is no sound evidence about the best timing to start the gliflozins therapy. In general, gliflozins (e.g., dapagliflozin and empagliflozin) are highly recommended drugs in all patients with reduced LVEF, with a level of recommendation of IA (McDonagh et al., 2021). However, in the main trials (DAPA-HF and EMPEREOR-Reduced) that led to the introduction of both dapagliflozin and empagliflozin in the guidelines for the treatment of HFrEF, gliflozins had been administered in patients with NHYA class > II, diabetic and not diabetic, in addition to optimal medical therapy with BB, ACEi/ARNI, and MRA. Gliflozins were, therefore, considered a ‘second-line’ treatment (McMurray et al., 2019; Packer et al., 2020). Moreover, a recent consensus from the American College of Cardiology suggests ARNI and BB as frontline therapies and MRA and SGLT2i as second-line treatments to be introduced in cases of persisting symptoms (Maddox et al., 2021). Our results support the use of gliflozins as frontline treatment as soon as a diagnosis of HFrEF is made, as delayed treatment would act in more advanced stages of cardiac remodeling, resulting in a lower clinical efficacy. As it occurs for other pharmacological therapies, the need to compare drugs in terms of efficacy and order of administration requires a continuous re-evaluation of the algorithm to use.

This study confirms how XAI and ML algorithms have the potential to go beyond the simplistic phenotyping of HF, e.g., HF with reduced or preserved ejection fraction. To date, this is the first observation of simple clinical parameters to predict CV response to gliflozins. Whether these predicting parameters are applicable to a larger population deserves further investigation. Furthermore, as gliflozins are effective in diabetic patients with HFpEF as well as in HF irrespective of the diabetic state (Anker et al., 2021; Packer et al., 2021; Peikert et al., 2022), it would be of interest to apply our analysis to additional populations.

### 4.1 Limitations of the study

Even if a significant number of features were described and collected for each patient and statistical robustness was assessed by cross-validation analyses, the number of patients enrolled is relatively small. The findings of our study should be interpreted with caution as being based on observational data, with potential unmeasured confounding and selection bias. Moreover, all patients have been enrolled in a single center. Nevertheless, despite the lack of an independent test set that prevented us from assessing the generalization power of the proposed models, our findings showed statistically significant differences between patients treated with gliflozins and those treated with standard treatment.

In conclusion, an ML analysis of a population of diabetic patients with HFrEF shows that SGLT2i treatment results in a beneficial effect in terms of LV remodeling, LV diastolic function, and biventricular systolic function. This cardiovascular response may be predicted by routine echocardiographic and clinical parameters with an explainable ML approach. The results will be further validated in larger populations.

## Data Availability

The raw data supporting the conclusions of this article will be made available by the authors, without undue reservation.
